# What Does Experience Teach to Be the Best Material for Filling Teeth?

**Published:** 1874-11

**Authors:** C. M. Wright

**Affiliations:** Basel, Switzerland


### DENTAL REGISTER.

VOL. XXVIII.] NOVEMBER, 1874. [NO. 11.

### "WHAT DOES EXPERIENCE TEACH TO BE THE BEST MATERIAL FOR FILLING TEETH?"

*Read before the American Dental Society of Europe, in Geneva, July 2d,* 1874.

BY C. M. WRIGHT, BASEL, SWITZERLAND.

This as you know is the third of the excellent questions propounded by the distinguished gentlemen who act as Executive Committee for the American Dental Society of Europe, and this is the subject I had concluded to illustrate and exhaust and present to your honorable body at this meeting.

I seized a golden oportunity, when the world around slept in quietude, when the office bell was resting from a day's and week's work, when all nature was hushed in a soft, summer repose that seemed to invite quiet thought and when my mind was disposed for a consultation with the Past, or Experience.

I placed ink-stand, pen-wiper, plenty of paper before me, washed my hands, trimmed my nails, took up my best pen, drew imaginary scrolls and lines of beauty in the air, at the same time moving my fingers airily to prove that physically, I was ready to let the ink flow as rapidly as my thoughts. The subject itself was written or copied, and I was stopped by the question, what or whose experience is meant in the question propounded. Shall I give my own experience and the results? Shall I state the material that has proved best in my own hands, or shall I judge from what I have observed of the work of other hands? When we ask what experience teaches we want facts; where shall we look for these facts that answer this question?

Once upon a time in the old Ohio Dental College there came *not* a solitary horseman, but a tall stooping, lounging, student of some ten or twelve years' practice in a solitary manner in some unknown rural region of the State of Ohio. His lack of reverence for young professors, his easy habit of sitting with his chair tipped back against the wall, and asking a spruce young professor, "What do you fellers consider the best material for filling teeth?" and similar actions and remarks hurt the dignity of some professors and induced sneers of contempt to appear on the faces of his fellow students. He was deemed a fit subject for buts and practical jokes, but this student proved to be a wonderfully apt scholar, a well read well versed man, a man of close observation and good judgment about his profession. His examination won the admiration of the professors of anatomy, physiology, pathology and chemistry. This man remarked that he had traveled a good deal through the country and had noticed that many more teeth were saved by the use of amalgam than with gold. He said he used a great deal of amalgam and believed in it, and dental students elevated their noses and walked away, they could not bring themselves to recognize a man who used amalgam or at least one who would say he used amalgam.

This remark however badly received by the students, caused me to reflect, to consider the question; and I went back to the days of my pupilage and remembered as well as I could about the amalgam and the gold I had all along seen in the different mouths, I remembered my old preceptor and his beautiful gold fillings, made with infinite patience, with infinite labor, and his method at that time of using gold in all ordinary and simple cavities and of using amalgam in difficult places, posterior surfaces of teeth, in broken down molars, etc., and I remembered how much pains he took in the preparation of his amalgam, the preparation of the cavities, the finishing and polishing and after burnishing his amalgam fillings, and then the appearance of the mouths he had operated upon. Afterwards, in Cincinnati, I had the opportunity of seeing many of the teeth treated and filled by this gentleman, and I think I have never seen so few failures from any dentist. I have seen amalgam fillings that have preserved the teeth well for twenty or thirty years in, positions that must have been badly broken down, from the hand of this same gentleman. I knew of course how conscientiously the work had been done. The gold fillings were also first class, but as I said not in the same difficult positions and cavities. Now this gentleman is a skillful operator, is known and has been known as a skillful operator. Judging from this observation of one man's work, we might say gold will preserve the teeth in accessible and not too large cavities when well introduced, and amalgam will preserve the teeth in badly broken teeth and in posterior approximal positions.

This of course is not the whole question. I know another distinguished operator, who will not use amalgam at all, and in many of these kinds of cases where my preceptor was cautious and sure and used amalgam this distinguished operator has employed gold. This gentleman does elegant work, makes what we properly call brilliant operations upon the teeth, and I have seen many failures from his hands; at the same time, I have seen really brilliant successes after three or four years standing. Can we deduce anything from this?

Shall we say "we may expect a proportion of failures from gold in difficult places?"

One chemist, or dental chemist, makes our bones ache, causes us to quake with shaking palsy, salivates us, poisons us, when we read his objections, chemical and physiological, to amalgam in the mouth, and we feel like going to the first dentist who has a Morrison engine and having the little amalgam filling bored out before we die and rot from its bad effects. Another chemist, dental chemist makes light of these objections and gives formulas for amalgams and we let our little amalgam filling remain.

In my own experience and observation I am not physician enough, nor scientific enough to distinguish the diseases produced by amalgams, but the *theory may* be good against it, therefore I am and have been sparing of its use. I do not care for the thunders of the amalgam haters, I *use* it when I want to. We can not take the opinion of crusaders at par on the amalgam, the whisky, or the tobacco and coffee questions, and it may be well in the use of amalgam to treat it as we do tobacco. "Yes you say, the *theory* is good against its use, but as for me, give me a little of the poison every day, if you please sir;" or, "Certainly madam, coffee is very bad: I'l thank you for another cup." I do not give these themes against tobacco, beer, tea, coffee as mine, for I do not know the physiological effects, though authority A. says one thing and authority B. says quite the contrary; and in these, like many other matters, science is simply a word that has been used so long and so often without a clear notion of its exact meaning that authorities A. and B. can claim its possession and parties are formed holding diverse opinions and *both* possessing science. In this place it may be proper to repeat a remark made to me by one of the editors of a German publication, the other day, viz: "The German dentists are doing more and are far ahead of the American in *science.*" I suppose I understood what was meant, but the word *science* is not a very clear one as it is commonly used; and German dentists, I might add, with their science use amalgams with great freedom; some of the scientific ones using it to the exclusion of any other metal. In this country there is a great deal of amalgam used and much of it is used with great carelessness, still the number of teeth saved is great. I have seen mouths full of ink-black amalgam that had been simply plastered in evidently, and have found no fresh decay. Is gold, then, the best material, or is amalgam by the light of experience to be used for filling teeth?

We all know the objections to gold in its use. We all know how much easier a plaster filling can be applied to the inequalities of a cavity. We all know how much purer and more elegant in appearance gold is when the surface is well polished and fine, and yet in all positions, to work the gold so that it may for a number of years retain the fine surface, is an operation our most skillful dentists do not claim as invariable in result. In moderately easy cases, cases that become easy to ordinary skill I mean, gold no doubt from its incompatibility is at present the best known material for filling teeth; but amalgam and tin foil, among the metals, will also, if as carefully used, save the teeth. Lead will also preserve teeth according to old stories, but is too base a metal for the mouth of course. Amalgam, in the hands of a dentist not very high in manipulative ability, will save teeth better than gold in the same hands; and, I presume, if we could get accurate statistics, we should find in Europe and America really very many more teeth saved for ten years by amalgam than by gold. Our privilege should be to select the material best suited to the case. In my experience the gutta percha fillings and the oxy-chloride of zinc fillings are temporary stoppings. They certainly have their places, but we are compelled at present to use metals. and the question is narrowed to two or three.

Amalgam turns black in most mouths and yet the chemists who are opposed to its nse, say it is less injurious after this oxydization than when bright. I could repeat or quote ob- objections in very scientific terms, but it will not be necessary before so learned a body. If the question is still more contracted and becomes one between soft and unannealed gold and annealed gold, I think we can safely say either will preserve teeth when properly inserted; and I will, if you please, repeat here a record of some experiments I made after reading the article in the April No. of Johnston's Dental Miscellany, entitled, "An unexpected Property of Adhesive Gold." (As these experiments were published in July in Johnston's Miscellany, we will not repeat them.)

Then added to these experiments might be one entitled "a very good quality of non-adhesive gold, viz, one of these same bits of ivory, with a regular cavity drilled into it, was dipped into water and while the cavity was full of water the gold was introduced in cylinders and wedges, in displacing the water afterwards it was found that the colored fluid could not penetrate the gold nor get in at the margins. I have done this occasionally in children's teeth in spite of the cry "keep your cavities dry," when from supersensitiveness of the mucus membrane of the mouth the patient could not bear the napkin or the rubber dam or bibulous paper and when the mouth overflowed. I have marked these cases on my record as sub marine operations etc., and have yet to find a larger proportion of failures than in the dry method. I have in fact no failures to report. In these cases the cavities are adapted for wedging the gold of course, and are neither contour or irregular in result or form. From this we might infer that an elective or an eclectic practice is allowable, and that while at 8 a. m. we use annealad foil or pellets, beginning from a retaining pit, and adding on our little pellets or mats carefully and with absolute dryness; at 10 a. m. we may in a different case and for a different patient, wipe out our cavity clean and without a napkin or bit of bibulous or a coffer dam, set up cyllnders or blocks and wedge them in tight and still do what we intend, viz, save the tooth from caries in this particular place. But some who want to argue a question might ask, "If the filling is good when introduced in this case at 10 a. m. why annoy your patients or spend your own time in adjusting the dam or keeping out moisture in other cases?"

Gold works much better dry, even soft gold; and with adhesive gold of course the operation is impossible as gold will not weld under water and as adhesive gold will make a cavity water tight, and as corners, contours and irregularly shaped cavities can be better filled when the gold is adhesive and capable of being made a unite; therefore we must in these cases keep out moisture and even in a majority of regular plain cavities where soft foil is admissible, dryness is a great advantage in hastening the operation, in allowing a more perfect view of the work, and in the superior neatness of the operation. Lately I had a lady patient who could not bear the slightest touch of napkin, finger or instrument to the palate; and, after several unsuccessful attempts with the dam, the napkin and paper, handled as gently as I possibly could, laid them all aside, and wiping out the cavity with spunk, introduced the gold regardless of moisture, and when finished I could not detect by the most thorough examination any objections either in finish or solidity or perfect margins to the soft foil fillings.

As dentists. with our own heads and hands to rely upon, we should be more independent of watch-words, of hackneyed phrases, etc. We should respectfully decline to sneeze when the great Dr. — sneezes, unless we are positive of the sensations ourselves of a strong draught. We should decline to say, "Gold is the best material for filling teeth," or "Amalgam is a poisonous compound for the mouth," or "Amalgam will not preserve the human teeth," or "Tin foil is next best to gold," or "Oxychloride of zinc is only a temporary filling," or any other of the common expressions, and there are hundreds of these in all departments of science, unless we with our own good heads and our own true hands have found out the fact. We are guilty of this fault, my brethren, and so are other professions; but this should not excuse us. When bromide of potassium and hydrate of chloral were introduced as medicines by some authority, I had the privilege of looking over a long file of prescriptions given by many prominent city physicians, and more than four-fifths of the prescriptions contained bromide of potassium and the hydrate of chloral, so that four-fifths of the diseases of men, women and dear little children; four-fifths of the ills that flesh is heir to, from gouty old bummer to the little boy with a stubbed toe, four-fifths, I say, of all these troubles were to be counteracted by the popular remedies of the day; bromide of potassium and hydrate of chloral. We dentists laugh at this the trade of medicine, but let us look to our trade, and what we know, that let us know from our heads and hands, our own powers of judgment, observation, comparison, and what we have not made ours by these faculties, let us acknowledge as not our property.

